# Can perceptual indices estimate physiological strain when wearing personal protective clothing in the heat?

**DOI:** 10.1186/2046-7648-4-S1-A144

**Published:** 2015-09-14

**Authors:** David Borg, Ian Stewart, Joseph Costello

**Affiliations:** 1 Institute of Health and Biomedical Innovation, Queensland University of Technology, Brisbane, Australia

## Introduction

Explosive ordnance disposal (EOD) often requires technicians to wear multiple protective garments in challenging environmental conditions. The accumulative effect of increased metabolic cost coupled with decreased heat dissipation associated with these garments predisposes technicians to high levels of physiological strain. It has been proposed [1] that a perceptual strain index (PeSI) using subjective ratings of thermal sensation and perceived exertion as surrogate measures of core body temperature and heart rate, may provide an accurate estimation of physiological strain. Therefore, this study aimed to assess if the PeSI could estmate the physiological strain index (PSI) across a range [2] of metabolic workloads and environments while wearing heavy EOD and chemical protective clothing.

## Methods

Eleven healthy males wore an EOD and chemical protective ensemble while walking on a treadmill at 2.5, 4 or 5.5 km.h^-1 ^at 1 % grade in environmental conditions equivalent to wet bulb globe temperatures of 21 °C, 30 °C or 37 °C. Trials were ceased at a maximum of 60 min or until the attainment of termination criteria. A Pearson's correlation coefficient, mixed linear model, absolute agreement and receiver operating characteristic (ROC) curves were used to determine the relationship between the PeSI and PSI.

## Results

**Figure 1:**
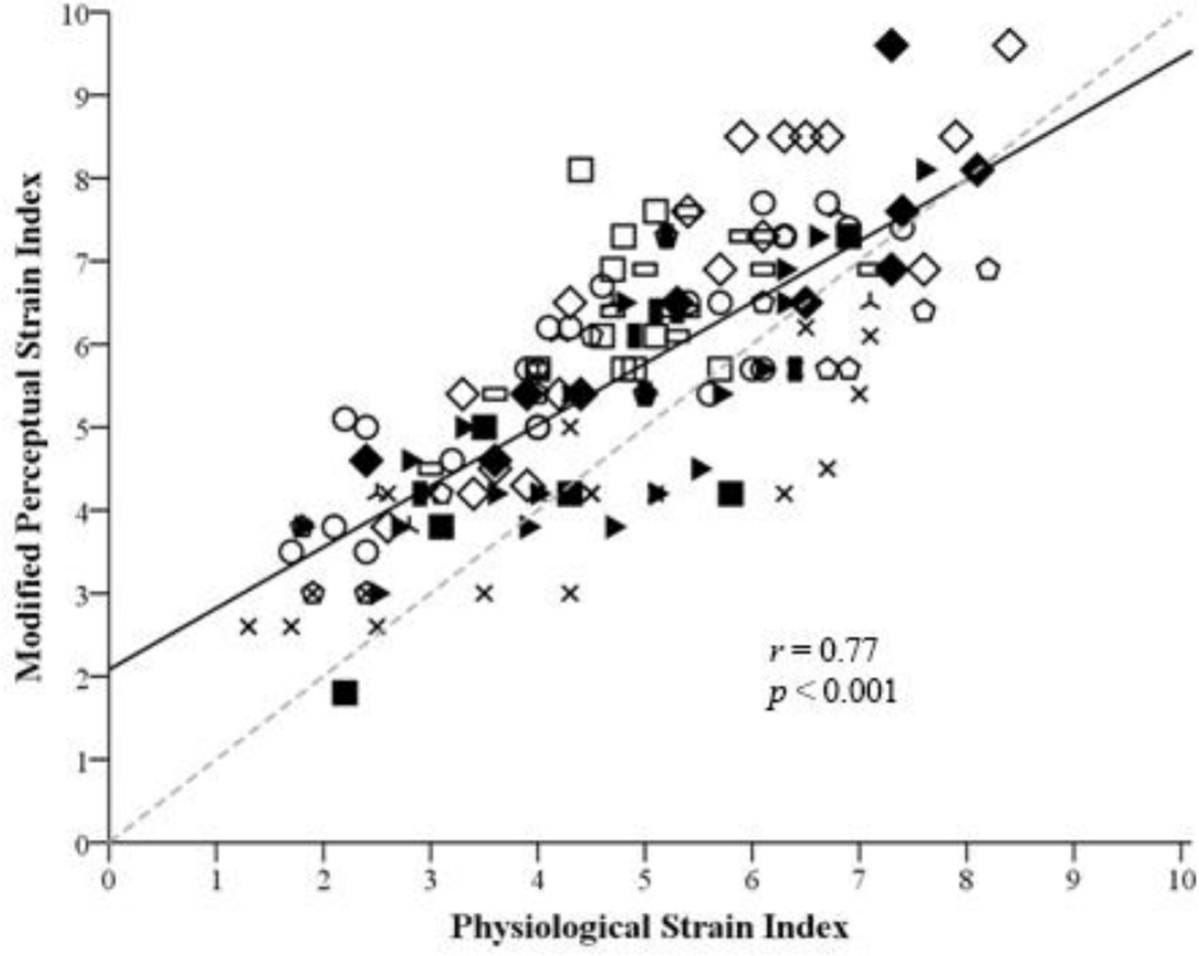
**Regression of the PSI and the PeSI for all participants across all trials and time points**. Solid line represents the trend line; each participant is represented by a unique symbol; the dashed line represents the line of identity.

A significant moderate relationship (Figure 1) between the PeSI and the PSI was observed [*r *= 0.77; *p *< 0.001; mean (SD) difference = 0.8 (1.1) a.u. (modified 95% limits of agreement -1.3 to 3.0)]. The ROC curves indicated that the PeSI had a good predictive power when used with two, single-threshold cut-offs to differentiate between low and high levels of physiological strain (area under curve: PSI three cut-off = 0.936 and seven cut-off = 0.841).

## Discussion

This is the first study to examine the ability of a PeSI to estimate physiological strain across a range of workloads and environments while wearing heavy protective clothing. The primary findings to emerge from this research are: (1) a statistically significant moderate relationship exists between the PeSI and PSI; and (2) the PeSI correctly or conservatively (over) estimated physiological strain 94.7% of the time.

## Conclusion

These findings support the use of the PeSI for monitoring physiological strain whilst walking and wearing EOD and chemical protective clothing. However, future research is needed to confirm the validity of the PeSI for active EOD technicians operating in the field.
